# Rac GTPase activating protein 1 promotes gallbladder cancer via binding DNA ligase 3 to reduce apoptosis

**DOI:** 10.7150/ijbs.58857

**Published:** 2021-05-27

**Authors:** Rui Bian, Wei Dang, Xiaoling Song, Liguo Liu, Chengkai Jiang, Yang Yang, Yongsheng Li, Lin Li, Xuechuan Li, Yunping Hu, Runfa Bao, Yingbin Liu

**Affiliations:** 1 Department of Biliary-Pancreatic Surgery, Renji Hospital, School of Medicine, Shanghai Jiao Tong University, Shanghai, 200127, China.; 2 Department of General Surgery and Laboratory of General Surgery, Xinhua Hospital, School of Medicine, Shanghai Jiao Tong University, Shanghai, 200092, China.; 3 Shanghai Key Laboratory of Biliary Tract Disease Research, Shanghai 200092, China.; 4 State Key Laboratory of Oncogenes and Related Genes, Shanghai Cancer Institute, School of Medicine, Shanghai Jiao Tong University, Shanghai, 200240, China.

**Keywords:** RACGAP1, LIG3, gallbladder cancer, DNA damage repair, apoptosis

## Abstract

Rac GTPase activating protein 1 (RACGAP1) has been characterized in the pathogenesis and progression of several malignancies, however, little is known regarding its role in the development of gallbladder cancer (GBC). This investigation seeks to describe the role of RACGAP1 and its associated molecular mechanisms in GBC. It was found that RACGAP1 was highly expressed in human GBC tissues, which was associated to poorer overall survival (OS). Gene knockdown of RACGAP1 hindered tumor cell proliferation and survival both *in vitro* and *in vivo*. We further identified that RACGAP1 was involved in DNA repair through its binding with DNA ligase 3 (LIG3), a crucial component of the alternative-non-homologous end joining (Alt-NHEJ) pathway. RACGAP1 regulated LIG3 expression independent of RhoA activity. RACGAP1 knockdown resulted in LIG3-dependent repair dysfunction, accumulated DNA damage and Poly(ADP-ribosyl) modification (PARylation) enhancement, leading to increased apoptosis and suppressed cell growth. We conclude that RACGAP1 exerts a tumor-promoting role via binding LIG3 to reduce apoptosis and facilitate cell growth in GBC, pointing to RACGAP1 as a potential therapeutic target for GBC.

## Introduction

Gallbladder cancer (GBC) is the most common malignancy of the biliary tract system. While this disease is relatively rare, it is associated with a dismal prognosis 1. Currently, the most effective regimen for GBC is surgical resection, however, the optimal time for curative removal is missed in the majority of patients due to a delay in diagnosis 2. Even in cases undergoing radical resection, recurrence occurs with either rapidly local or distant metastases. The response rate to standard chemotherapy for advanced unresectable GBC is still unsatisfactory, and no effective targeted agent against GBC is currently available outside of clinical trials 3. More reliable biomarkers and therapeutic targets are necessary in improving early diagnostic rates and treatment efficacy.

Rho GTPases are classified as the members of the Ras superfamily and control various cell processes through molecular switches between active GTP-bound state and inactive GDP-bound state 4. Rac GTPase activating protein 1 (RACGAP1), which is also referred to as MgcRacGAP and CYK4, is originally categorized as a type of GTPase activating proteins (GAPs) that stimulate intrinsic activity of Rho GTPases and enhances GTP hydrolysis 5. RACGAP1 is a well-documented modulator of cytokinesis, migration and differentiation. In addition, increasing evidence reveals that RACGAP1 contributes to tumorigenesis and malignant progression of several malignancies, such as hepatocellular carcinoma 6, bladder cancer 7, gastric cancer 8, and breast cancer 9. The activity of RACGAP1 characterized with Rho GTPases is involved in exerting effects in these malignant tumors, however, its functional analyses indicated that its activity is not limited to Rho GTPases. Instead, additional specific signature of RACGAP1 was emerging 10. Therefore, our study aims to characterize the role of RACGAP1 in GBC and its regulatory mechanisms.

Each human cell could experience more than 10,000 DNA lesions per day, an event which is typically triggered by normal cellular processes. Severe DNA damage causes cell apoptosis, and DNA alterations are risk factors of cancer and age-related diseases 11. Cell responses to DNA damage rely on complex mechanisms, in which DNA ligases catalyze the process of recruiting a large number of enzymes and proteins to the end-joining of DNA strands 12, 13. DNA ligase 3 (LIG3) is an important molecule in the alternative-non-homologous end joining (Alt-NHEJ), a pathway that possesses a high error rate in repairing DNA double-strand breaks (DSBs), leading to cell survival from DNA damage but with the unavoidable adverse effect of genomic instability 14. LIG3 downregulation or its failure to localize at the site of DSBs contributes to dysfunction of Alt-NHEJ pathway, which is noted to reduce DNA repair efficiency, resulting in apoptosis and growth retardation in a number of malignant tumors 15, 16, 17.

Herein, we uncovered that higher RACGAP1 level correlates to poorer overall survival in patients with GBC. The binding between RACGAP1 and LIG3 makes DNA damage repair involved in regulation of GBC cells viability.

## Materials and Methods

### Tissue specimens and cell lines

Two separate cohorts of tissue specimens were collected from the Department of General Surgery, Xinhua Hospital, Shanghai Jiao Tong University School of Medicine (Shanghai, China). Cohort#1 contained 50 GBC and 50 cholecystitis samples from sample bank of pathology department, all formalin-fixed and paraffin-embedded, used for IHC and microscopical examination. Cohort#2 contained 32 samples of paired fresh-frozen GBC and corresponding adjacent tissues from patients who underwent radical cholecystectomy, used for RNA extraction. Patients who were treated preoperatively with chemo- or radiotherapy were excluded from this study. Each specimen was pathologically staged in accordance with the AJCC 8^th^ edition of TNM Classification of Malignant Tumors. Informed consent was documented from each patient, with the Ethics Committee of Xinhua Hospital providing approval for the study protocols.

The human gallbladder epithelial cells (HGEpC) was previously established and characterized by the Shanghai key laboratory of biliary tract disease research. GBC cell lines GBC-SD were procured from the Cell Bank of Shanghai Institutes for Biological Sciences, Chinese Academy of Sciences (Shanghai, China). NOZ, SGC-996 and OCUG-1 cell lines were purchased from the Health Science Research Resources Bank (Osaka, Japan). EH-GB1 was a present from Shanghai Eastern Hepatobiliary Surgery Hospital. DMEM medium (Gibco, NY, USA) was used to maintain all cells with the addition of 10% fetal bovine serum (Gibco, NY, USA). Culture environments were composed of 5% CO2 and were maintained at 37 °C.

### Immunohistochemistry (IHC) and scoring

IHC staining was performed using standard immunoperoxidase staining procedures. A RACGAP1 monoclonal antibody (Abcam, MA, USA) was diluted to the ratio of 1:200. RACGAP1 expression levels were semi-quantitated through integration of the proportion of positive tumor cells and the positive staining intensity as Xiang S et al. described 18. The proportion of positive tumor cells was scored as 0 (0% positive cells), 1 (≤10% positive cells), 2 (11%-50% positive cells), 3 (>50% positive cells). Staining intensity was graded as follows: 0 (negative), 1 (weak), 2 (moderate), and 3 (strong). The sum of staining intensity and positive proportion were recorded as the final immunoreaction score, ranging from 0 to 6. Samples were then cohorted as follows: negative (0), weak (1-2), moderate (3), and strong (4-6) staining. Scores above or equal to 3 was marked as samples with high RACGAP1 expressions while those below this was designated as samples with low RACGAP1 expressions.

### Plasmids and regents

Small Interfering RNAs (siRNA) were designed by Biotend Company (Shanghai, China), and short hairpin RNAs (shRNA) were synthesized by GenePharma Company (Shanghai, China). We used a mixture of three siRNAs towards specified gene. The siRNA sequences were listed in Table S1. The RFect Reagent (Changzhou Biogenerating Biotechnologies corporation, China) was used to transfect siRNA into cells in compliance to protocols stipulated by the manufacturer. Era Biotech (Shanghai, China) was employed to clone full cDNA length of the specified genes.

Cycloheximide (CHX) and etoposide was purchased from MedChemExpress (NJ, USA). Cytoskeleton (CO, USA) supplied the Rho inhibitor I (CT04). Cells were treated with 2 μg/mL CT04 for 24 hours. 10 μg/mL Etoposide was added to the cells and the mixture was cultured for 2 hours. The working concentration of CHX used was 50 μg/mL.

### Cell proliferation assay

The Cell Counting Kit-8 (Yeasen Biotech, Shanghai, China) was used to assess cell viability. NOZ, GBC-SD and SGC-996 cells were cultured in 96-well plates at a concentration of 1,000 cells per well. CCK-8 working solution was prepared with culture medium and CCK-8 solution (v/v=10:1). Culture medium was replaced with 100μL working solution per well and subjected to a 2 hour incubation period at 37 °C. A microplate reader was used to construct cell proliferation curves based on absorbance at 450 nm.

### Colony formation assay

6-well plates were used to seed 500 cells/well which were pre-treated with the indicated regimen. The cells were allowed to culture for 10 days. This was followed with cell fixation using 4% paraformaldehyde for 15 min. Cells were then exposed to 0.1% crystal violet for 15 min at room temperature. We then quantified colonies before the cells were imaged.

### Cell apoptosis and TUNEL assay

Cell apoptosis was detected using the FITC Annexin V Apoptosis Detection Kit (BD Biosciences, CA, USA) based on instructions stipulated by the manufacturer. 6-well plates were used to culture pre-treated cells for 48 hours. Cells were harvested and resuspended in 100 μL binding buffer containing 5 μL FITC-conjugated annexin-V and 5 μL propidium iodide (PI). After incubation, the cell suspension was exposed to 400 μL binding buffer before being analyzed with flow cytometry to assess the degree of cell apoptosis.

TUNEL assays of tumor tissues were performed using TUNEL Apoptosis Detection Kit (DAB) (Beyotime, Shanghai, China) as manufacturer's protocol.

### Cell cycle analysis

NOZ and GBC-SD cells were harvested and fixed in cold 70% ethanol overnight at 4 °C. Then, cells were incubated in 500 μL of 1mg/mL PI solution containing 10 mg/mL RNase A for 30 min at 37 °C in the dark. Cell cycle phase of all samples were evaluated with flow cytometry.

### Xenografted animals

Female nude mice of ages 4-6 weeks old were obtained from the Shanghai Laboratory Animal Center of the Chinese Academy of Sciences (Shanghai, China), and reared under standardized conditions in compliance to institutional animal care protocols. NOZ cells were infected with lentivirus expressing shRNA or/and specified gene before being subjected to antibiotics selection. 1 × 10^6^ of stably infected NOZ cells were inoculated subcutaneously into the armpits of mice (5 mice per group). Weekly observations of tumor growth were documented, with three-dimensional measurements made. Tumor volumes were derived based on the following formula: tumor volume = π / 6 × width × length × height. At the end of the experimental period, the mice were sacrificed, and the tumors were harvested and weighed.

### Quantitative real-time PCR (qRT-PCR)

TRIzol reagent (Takara, Shiga, Japan) was used to isolate total RNA samples based on instructions provided by the manufacturer. 1 ug of RNA was used to synthesize cDNA with PrimeScript^™^ RT regent Kit with gDNA Eraser (Takara, Dalian, China). TB Green^®^ Premix Ex Taq™ (Takara, Shiga, Japan) and a StepOne Plus system (Applied Biosystems, CA, USA) were used to carry out qRT-PCR. The sequences of primers were listed in Table S2. The 2^-ΔΔCt^ method was used to calculate the relative expression of each gene.

### Western Botting and antibodies

The Radio Immunoprecipitation Assay (RIPA) buffer (Beyotime, Shanghai, China) containing 1% protease inhibitor cocktail (Beyotime, Shanghai, China) was used to extract cellular protein. The Nuclear and Cytoplasmic Protein Extraction Kit (Beyotime, Shanghai, China) was use to extract subcellular protein. The extracted samples were subjected to sodium dodecyl sulfate polyacrylamide gel electrophoresis in order to separate the component proteins before they were immunoblotted onto PVDF membranes. 5% nonfat dry milk in TBST was used to block membranes. The appropriate primary antibodies were then added to the membranes and allowed to incubate. This was followed by incubation with HRP-conjugated secondary antibodies. Western blotting analysis was performed using anti-RACGAP1, LIG3, γH2A.X, p-ATM, p-ATR, p-CHEK1 and p-CHEK2, PARP1, caspase3 and c-caspase3, PAR, GAPDH and Histone H3 antibodies (p-ATM, p-ATR, p-CHEK1, p-CHEK2 and PARP1 were supplied by ABclonal, MA, USA, others were from Abcam, MA, USA).

### Immunoprecipitation-Mass spectrometry assay (IP-MS)

A 10% acrylamide gel (ThermoFisher Scientific, MA, USA) was used to load protein samples. Sample lanes were excised after running, and the proteins in-gel were digested with trypsin and evaluated using MS-MS at the Shanghai Applied Protein Technology.

### Co-immunoprecipitation assay (Co-IP)

Co-IPs were performed with protein G magnetic beads (Bio-Rad, CA, USA) in accordance with the directions provided by manufacturer. Magnetic beads were first incubated with an anti-RACGAP1 or anti-LIG3 antibody for 4h, and then incubated with NOZ and GBC-SD cell lysates overnight at 4 °C. A lysis buffer was used to rinse the magnetic beads before they were assessed with western blotting.

### Immunofluorescence assay (IF)

GBC-SD and NOZ cells were seeded in 12 well-plates, which were previously laid with sterile cover glasses, and incubated for 24h before being stained. 4% paraformaldehyde was used for cell fixation which were then permeabilized in 0.2% Triton X-100 at room temperature. 0.1% bovine serum albumin (BSA) was then used to block cells prior to an overnight incubation with anti-RACGAP1 (1/400), anti-LIG3 (1/200) and anti-γH2A.X (1/200) at 4°C. This was then followed by an incubation at 37 °C with a Alexa Fluor 488 or Alexa Fluor 594 conjugated secondary antibody (Yeasen Biotech, Shanghai, China). DAPI was then used to counterstain the cells before a Leica microscope was used to image the cells.

### Commet assay

The Comet Assay Kit (Abcam, MA, USA) was used to perform the comet assay as instructed by the manufacturers. GBC cells were grown in 35-mm dishes transfected with specified siRNA. The following steps were performed in the dark. Harvested cells were suspended in ice-cold 1× PBS at a density of 10^5^ cells/ml. Cells were then mixed with comet agarose at 1/10 ratio (v/v), and immediately transferred onto slides coated with an agarose layer. Cells were then allowed to lyse in cold, freshly made lysis buffer for 1.5 h at 4 °C. The slides were then subjected to a 30 minute incubation period in alkaline solution at 4 °C. The cells were then transferred to a horizontal electrophoresis chamber filled with cold alkaline electrophoresis solution and electrophoresed for 25 min at 20 V. The slides were placed in pre-chilled DI H_2_O at room temperature, which was replaced every 2 min. Cells were then fixed with cold 70% ethanol, allowed to air dry, and dyed using vista green DNA dye for 15 min at room temperature to identify DNA tracks. The gel was evaluated using a fluorescence microscope with a FITC filter.

### Alt-NHEJ assay

The EJ2-GFP plasmid (Alt-NHEJ reporter) 19 was constructed by Era Biotech (Shanghai, China). The Alt-NHEJ assay was performed according to previously documented protocols 20. Cells were transfected with 2 μg Scel-linearized EJ2-GFP. Flow cytometry was then used to analyze these cells 72 hours later for GFP. 0.2 μg of DsRed expressing plasmid was co-transfected to normalize for any variations in transfection efficiency.

### Rho GTPase pulldowns

GTP-bound RhoA, Cdc42 and Rac1 levels were evaluated using the RhoA/Rac1/Cdc42 Activation Assay Combo Biochem Kit (Cytoskeleton, Denver, USA) as described in manufacturer's protocol.

### Statistical analysis

The Prism 8 (GraphPad Software) was used for all statistical analyses. Data from the experiments are depicted in terms of mean ± standard deviation (SD). All experimental data was a compilation of three separate experiments. Quantitative variables were assessed with the 2-tailed Student's *t* test. The Pearson's χ^2^ was used to analyze associations between RACGAP1 expression and clinicopathologic characteristics. Survival analysis was performed with Kaplan-Meier methods and the log-rank test. Statistical significance was recognized in results with *P* < 0.05. The *P* values are replaced with the following symbols in the figures: *P < 0.05, **P< 0.01, ***P < 0.001.

## Results

### RACGAP1 upregulation is associated with poor survival of GBC patients

**Figure 1:**
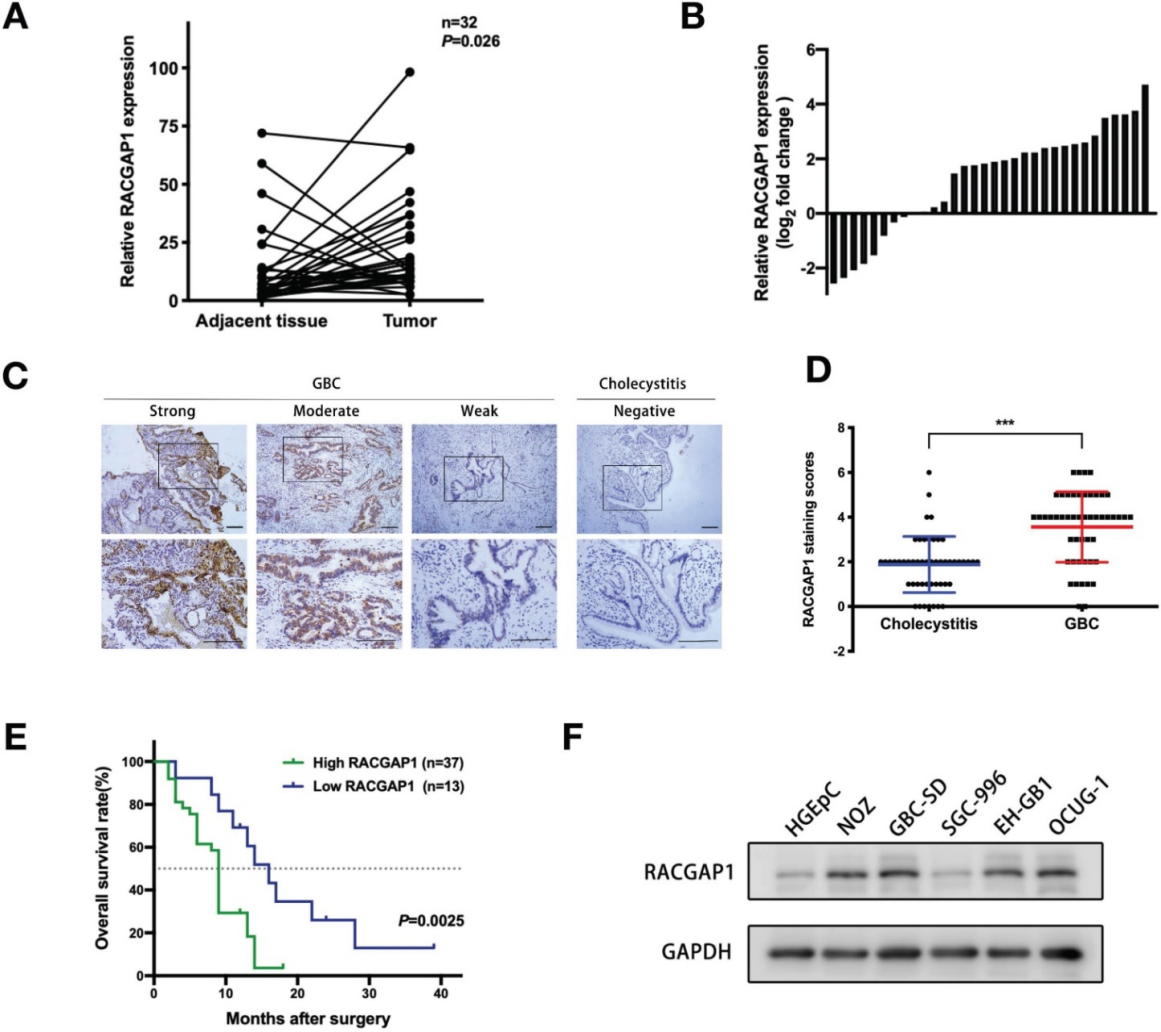
** Increased RACGAP1 levels are associated to poorer prognosis in patients with GBC.** (A-B) qRT-PCR evaluation of RACGAP1 expression in 32 pairs of GBC tumor tissues and adjacent normal tissues. (C) Representative IHC images of GBC and cholecystitis samples with an anti-RACGAP1 antibody. Scale bars represent 100 μm. (D) Scatterplots of the staining scores of RACGAP1 expression in patients with GBC and cholecystitis. (E) Kaplan-Meier overall survival curves based on RACGAP1 expression across GBC patients. The dotted line indicated cumulative survival=50%. (F) RACGAP1 expression in normal gallbladder epithelium cells and GBC cells. ***P < 0.001.

To determine the role of RACGAP1 in the pathophysiology of GBC, we first measured mRNA levels of RACGAP1 in 32 pairs of fresh GBC and matched adjacent normal tissues. RACGAP1 mRNA was markedly upregulated in tumor samples in contrast to matched controls (Figure 1A-B; *P* = 0.026). Immunohistochemistry (IHC) was next performed in 50 samples of GBC and 50 samples of cholecystitis as controls to evaluate protein level of RACGAP1. RACGAP1 was found to localize primarily in GBC cell nuclei. Analysis of stain scores confirmed that RACGAP1 was strongly upregulated in GBC tissues (Figure 1C-D) (Table 1).

We then analyzed correlations of available clinicopathological characteristics of GBC patients and RACGAP1 level. A higher RACGAP1 expression was linked to the larger tumor size (*P* = 0.015), deeper tumor invasion (*P* = 0.015) and the presence of gallstones (*P* = 0.026) (Table 2). The mean tumor size of high RACGAP1 group and low RACGAP1 group were 3.665±1.869 cm and 2.469±1.495 cm, respectively (*P*<0.05). In addition, we found that higher RACGAP1 staining scores were significantly associated with shorter overall survival (OS) in GBC patients (Figure 1E, *P* =0.0025), suggesting its pivotal contribution to GBC progression.

RACGAP1 protein levels were also assessed in normal human gallbladder epithelial cell line (HGEpC) and a panel of GBC cell lines. RACGAP1 expression was found to be elevated in four GBC cell lines (NOZ, GBC-SD, EH-GB1 and OCUG-1) in contrast to the low expression found in the HGEpC and SGC-996 (Figure 1F). The NOZ, GBC-SD, and SGC-996 cell lines were chosen for subsequent experiments.

### RACGAP1 promotes proliferation and repressed apoptosis of GBC cells *in vitro* and *in vivo*

**Figure 2:**
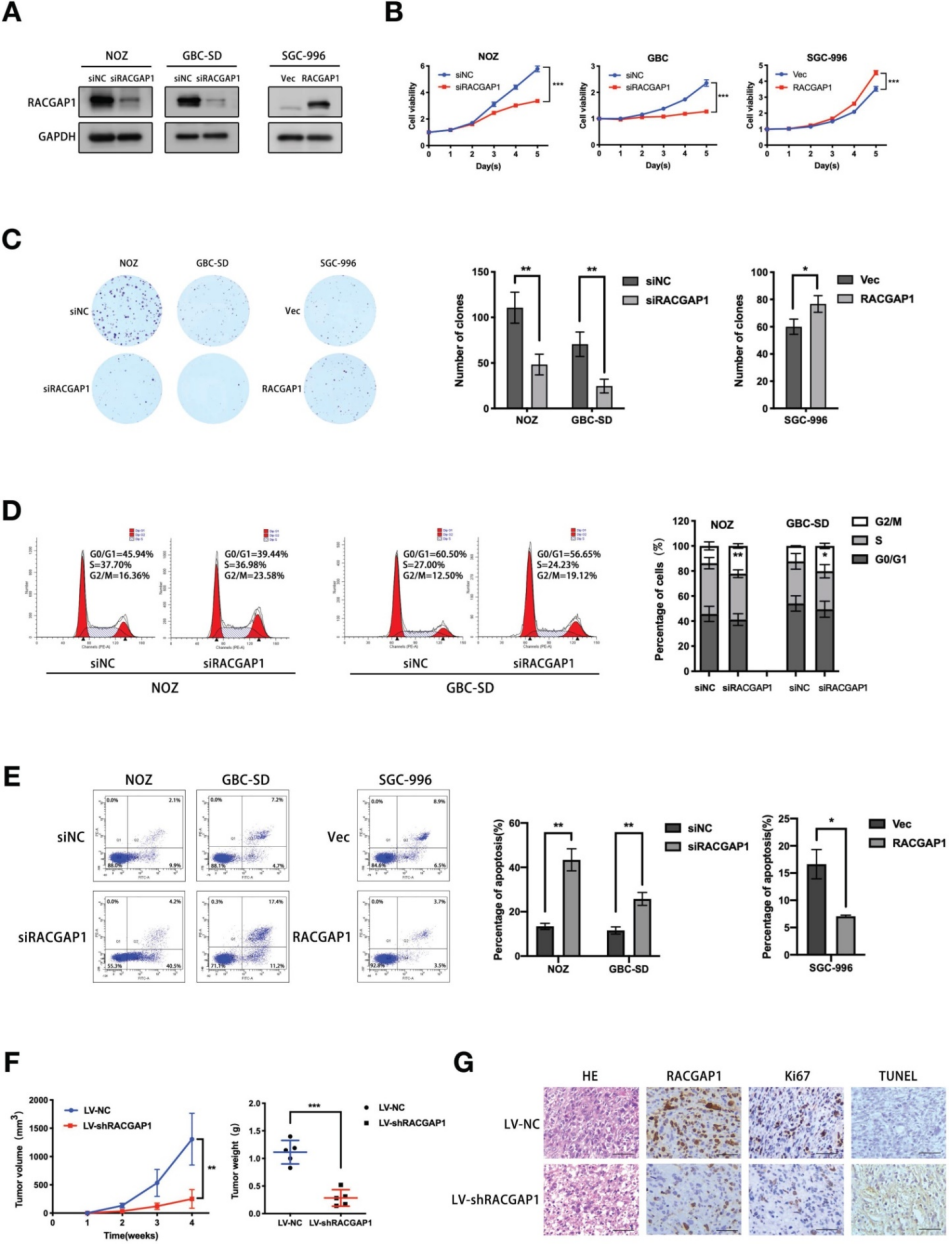
** RACGAP1 promotes growth and repressed apoptosis of GBC cells both *in vivo* and *in vitro*.** (A) Western blotting experiments of RACGAP1 expression in both GBC-SD and NOZ cell lines which were transfected with siRNA against RACGAP1 and in SGC-996 cell line with RACGAP1-expression vector. (B) Effects of RACGAP1 knockdown or overexpression on GBC cells viability by CCK-8 assays. (C) Colony formation assays performed on NOZ, GBC-SD and SGC-996 cells, with the number of colonies quantified and analyzed. (D) Effects of RACGAP1 knockdown on GBC cell cycle distribution were assessed using flow cytometry. (E) Effects of RACGAP1 knockdown or overexpression on apoptotic rates of GBC cells were assessed by flow cytometry. (F) Effect of RACGAP1 on the growth of subcutaneous tumors constructed by injecting NOZ cells transfected with lentiviruses. Tumor growth are depicted in the form of a line chart. Weight of tumors are depicted using scatterplots. (G) HE, RACGAP1, Ki-67 and TUNEL staining of the subcutaneous xenograft tumors from LV-NC and LV-shRACGAP1 group. Scale bars represent 50 μm. *P < 0.05, **P < 0.01, ***P < 0.001.

To clarify biological activity of RACGAP1 in GBC cells, knockdown experiments using siRNAs were performed in two GBC cell lines, NOZ and GBC-SD. siRNA-induced RACGAP1 suppression was validated by qRT-PCR and Western blotting (Figure S1, Figure 2A). Cells were then employed to evaluate proliferation and anchorage dependent colony formation ability with CCK-8 and colony formation assays. Both types of cell lines were susceptible to RACGAP1 knockdown, as evidenced by impaired of cell viability observed after RACGAP1 downregulation (Figure 2B). Consistent with this data, colony formation of RACGAP1 knockdown cells was suppressed by more than 50% of control cell colonies (Figure 2C). As expected, induced proliferation results were obtained in experiments involving RACGAP1 overexpression in SGC-996 (Figure S1, Figure 2A-C). We also performed cell cycle and cell apoptosis assays. Flow cytometry analysis showed that RACGAP1 downregulation could induce G2/M phase arrest and an increase of apoptotic cells (Figure 2D-E). Conversely, RACGAP1 overexpression resulted in resistance to apoptosis (Figure 2E), but did not affected significantly the cell cycle distribution of SGC-996 cells (Figure S2).

We proceeded to verify the* in vivo* role of RACGAP1 in GBC development. NOZ cells stably silencing RACGAP1 and control cells were transplanted subcutaneously into the armpits of nude mice. Consistent with *in vitro* data, RACGAP1 shRNA group displayed a significant tumor growth inhibition as volume and weight were only 20% and 25%, respectively, of those in the control group (Figure 2F, Figure S3). Moreover, IHC analysis showed that Ki-67 levels of shRACGAP1 tumors were lower than that of NC group, and compared with the NC group, we detected a higher level of apoptotic signal (TUNEL) in shRACGAP1 tumor tissues (Figure 2G).

### RACGAP1 binds with LIG3 in GBC cells

**Figure 3:**
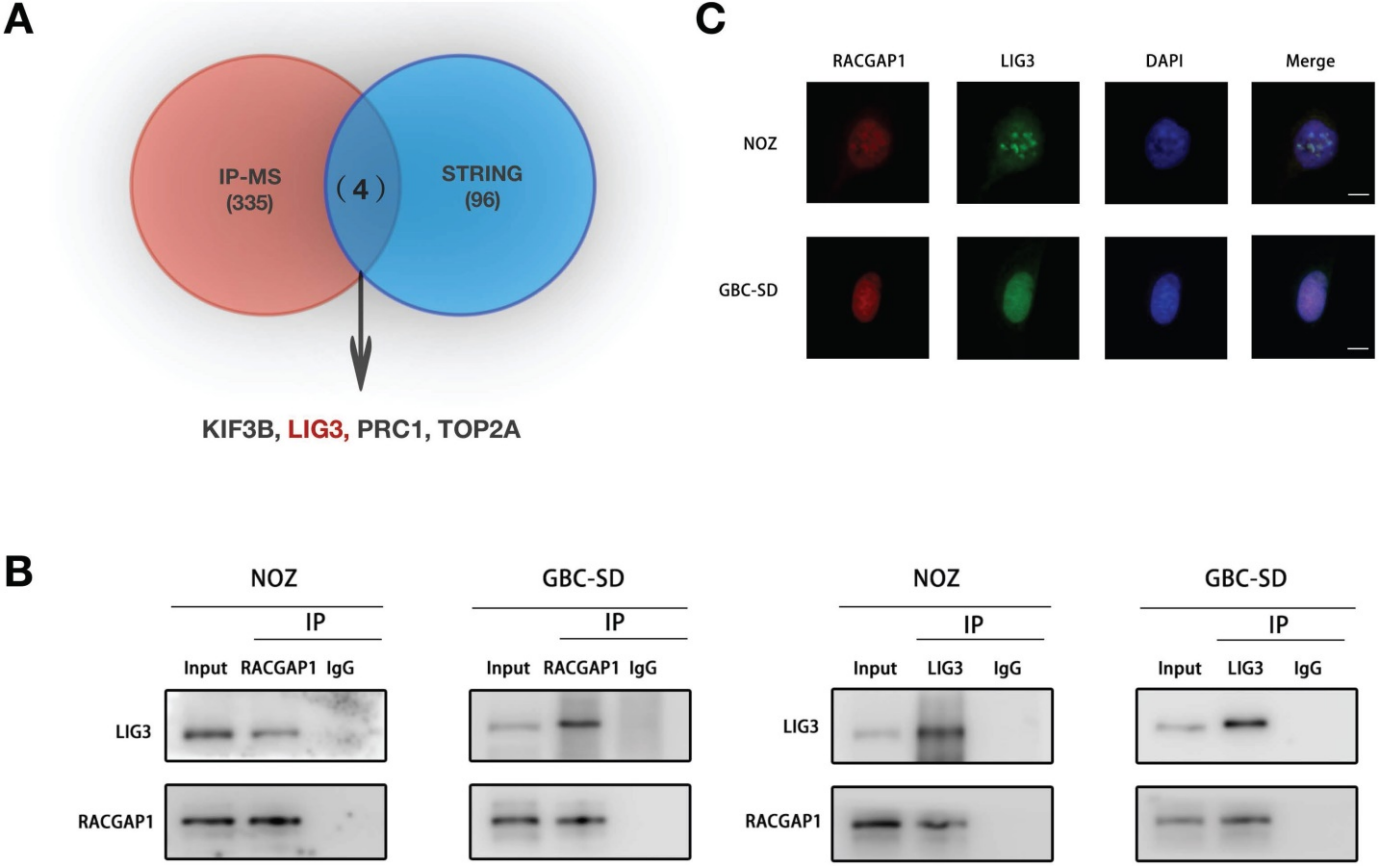
** RACGAP1 binds with LIG3 in GBC cells.** (A) A Venn diagram of potential RACGAP1 binding targets, as predicted using the STRING database and IP-MS. (B) Immunoblotting using antibodies as indicated after endogenous Co-IP with anti-RACGAP1 or anti-LIG3 antibody. (C) IF images of RACGAP1, LIG3 and DAPI in NOZ and GBC-SD cells. Scale bars represent 10 μm.

To unravel the molecular mechanisms underlying how RACGAP1 enhances viability of GBC cells, we performed immune-precipitation coupled to mass spectrometry (IP-MS) experiments that can identify the potential binding factors for RACGAP1. Using IP-MS whole proteomic analysis, we screened 339 binding candidates (UnquePepCount ≥3). We then searched the STRING database (Interaction score ≥0.900) and identified 4 putative factors (KIF3B, LIG3, PRC1 and TOP2A) from which the two screening algorithms were overlapped (Figure 3A). The Co-IP strategy was used to further detect the association between RACGAP1 and these proteins in GBC cells. The results of endogenous Co-IP showed that LIG3 bound to RACGAP1, and reverse Co-IP experiments validated the interaction (Figure 3B). The binding between RACGAP1 and PRC1 was also found in NOZ cells but not in GBC-SD cells, and Co-IP assays revealed that RACGAP1 did not interact with TOP2A and KIF3B (Figure S4). The cellular localization of RACGAP1 and LIG3 was confirmed by IF. As shown in Figure 3C, the RACGAP1 signal co-localized with the LIG3 signal primarily in the nuclei of NOZ and GBC-SD cells.

### RACGAP1 regulates LIG3 expression independent of RhoA activity

**Figure 4:**
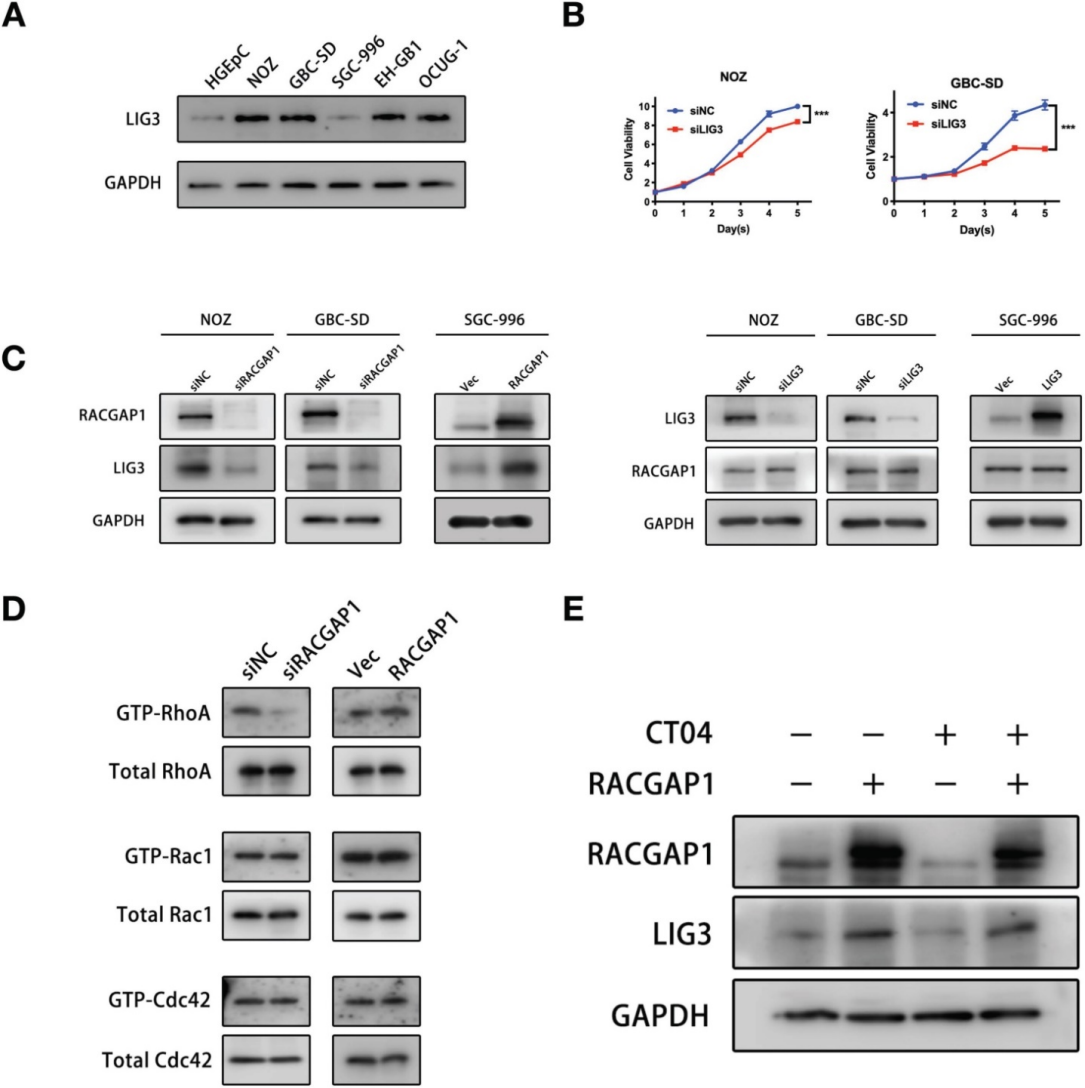
** RACGAP1 regulates LIG3 expression independent of RhoA activity.** (A) LIG3 expression in GBC cells and normal gallbladder epithelium cells. (B) Effects of LIG3 knockdown on GBC cells viability by CCK-8 assays. (C) Effects of RACGAP1 and LIG3 knockdown or overexpression on protein levels of each other in GBC cells. (D) Pulldowns in NOZ lines and immunoblotting assays of Cdc42, Rac1, and RhoA GTPase activity after RACGAP1 knockdown or overexpression. (E) Western blotting analysis of LIG3 protein level after RACGAP1 overexpression and Rho inhibitor I (CT04) treatment. ***P < 0.001.

We evaluated the expression level of LIG3 protein in GBC cell lines. We found, importantly, that LIG3 expression trend is the same as that of RACGAP1 in GBC cell lines, suggesting a positive correlation with RACGAP1 (Figure 4A). Furthermore, the CCK-8 assays revealed that suppressing LIG3 significantly reduced viability of GBC cells, and it induced appearance of apoptotic markers as well (Figure 4B, Figure S5B), suggesting that LIG3 plays a role in promoting GBC as well, which is consistent with RACGAP1. Meanwhile, we observed a mild viability inhibition of NOZ cells after PRC1 knockdown (Figure S5A). Then, we found that knockdown of PRC1 did not significantly affect the apoptotic markers of NOZ cells (Figure S5B), so its inhibition for NOZ viability maybe merely result from suppression of cell proliferation. Given the RACGAP1-binding and functional consistency of LIG3 in both NOZ and GBC-SD and its close correlation with RACGAP1 function and expression in GBC, we selected it for further study and hypothesized that RACGAP1 and LIG3 have a positive cooperation in mediating GBC cell viability.

In view of the correlation between the expressions of RACGAP1 and LIG3 in GBC cell lines, we exogenously altered the expression levels of one of the two to examine its impact on the expression levels of the other gene. Western blotting showed that RACGAP1 knockdown led to a marked decline in LIG3 protein levels, and augmented RACGAP1 expression induced LIG3 protein, whereas alteration of LIG3 protein levels did not affect RACGAP1 expressions (Figure 4C). These results suggested that LIG3 may function as a downstream factor of RACGAP1.

RACGAP1 is originally categorized as a protein that activates Rho GTPases. A critical question at this juncture is whether the activity of RACGAP1 with Rho GTPases is involved in the regulation of LIG3 expression. However, its target selectivity and specific effects on Rho GTPases has not been well established. Various studies have shown that RACGAP1 acted as a GAP towards Rac1 and cdc42 but does not exert the same activity towards RhoA 21, 22. It has also been revealed that RACGAP1 could be converted to RhoA-specific GAP by Aurora B 23. Others reported that RACGAP1 increased RhoA activity by stabilizing ECT2 6, 24. Our results showed that RACGAP1 knockdown suppressed and RACGAP1 overexpression enhanced RhoA activity while exerting little influence on the activities of Rac1 and Cdc42 in NOZ cells (Figure 4D). Suppressing RhoA activity using the Rho inhibitor I (CT04), did not reverse LIG3 augmentation triggered by RACGAP1 upregulation (Figure 4E), suggesting that RACGAP1 regulates LIG3 expression independently of RhoA activity.

### RACGAP1 downregulation increases DNA damage and apoptosis via LIG3 suppression

**Figure 5:**
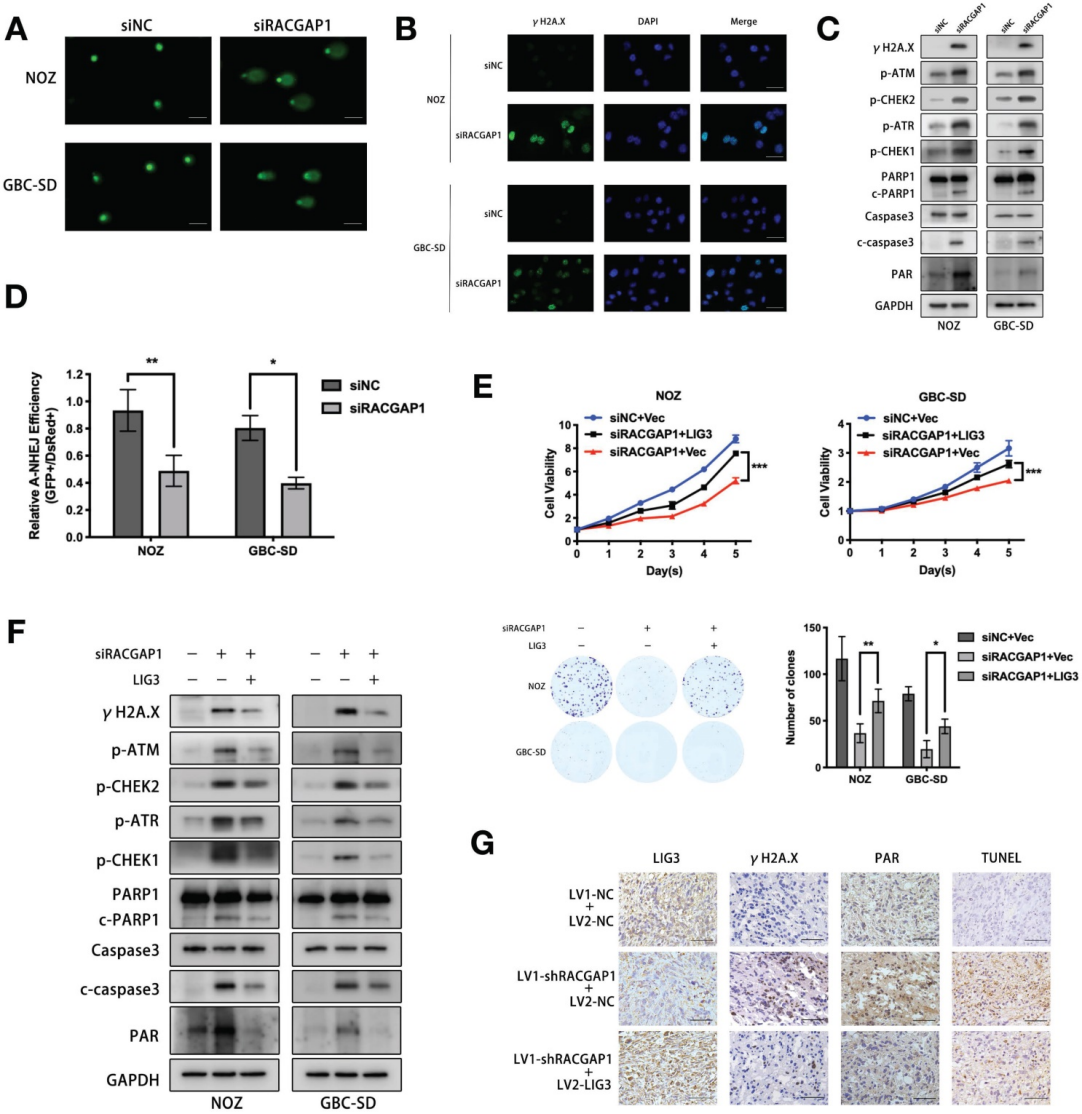
** RACGAP1 downregulation suppresses LIG3-dependent DNA repair which increases DNA damage and apoptosis.** (A) DNA damage after RACGAP knockdown in NOZ and GBC-SD cells was identified by the comet assay and observed with a fluorescence microscope. Scale bars represent 100 μm. (B) IF images of DSBs marker (γH2A.X) and DAPI after RACGAP1 knockdown. Scale bars represent 50 μm. (C) Western blotting detected the levels of γH2A.X, DDR (p-ATM, p-ATR, p-CHEK1 and p-CHEK2) and apoptosis (PARP1, c-PARP1, caspase3 and c-caspase3) related proteins, PAR signal in GBC cells after RACGAP1 knockdown. (D) Efficiency of Alt-NHEJ repair was evaluated by EJ2-GFP assay in NOZ and GBC-SD cells after RACGAP1 knockdown. (E) Cell viability and colony formation ability were evaluated in GBC cells transfected with siNC plus vector or siRACGAP1 plus vector or siRACGAP1 plus LIG3. (F) Western blotting analysis of γH2A.X, p-ATM, p-ATR, p-CHEK1, p-CHEK2, PARP1, c-PARP1, caspase3 and c-caspase3, PAR in GBC cells after transfection as indicated. (G) LIG3, γH2A.X, TUNEL and PAR staining of the subcutaneous tumors from xenograft models constructed with NOZ cells transfected with lentiviruses as indicated. Scale bars represent 50 μm.*P < 0.05, **P< 0.01, ***P < 0.001.

LIG3 is involved in DNA repair as a critical factor of the Alt-NHEJ pathway, and its deregulation or dysfunction impairs repair process 25. Our finding that LIG3 was regulated by RACGAP1 prompted us to examine whether RACGAP1 exerts its promoting effect on GBC through governing the DNA repair process. We performed the Alkaline comet assay which can assess the degree of DNA damage. As shown in Figure 5A, RACGAP1 depletion lengthened DNA tails and decreased head diameters. The observations suggested that RACGAP1 downregulation led to DNA damage in GBC cells. Double strand breaks (DSBs) are the most severe DNA damage which may induce cell apoptosis. After RACGAP1 knockdown, the frequency of DSBs increased markedly which was revealed by IF and western blotting for γH2A.X, a marker of DSB (Figure 5B-C), just as a rise of γH2A.X level that we observed after LIG3 knockdown in GBC cells (Figure S5B). In addition, RACGAP1 inhibition significantly activated DDR and caspase-dependent apoptotic signaling, as shown by raised levels of phosphorylated ATM, ATR, CHEK1, CHEK2, as well as caspase-3 and PARP1 cleavage (Figure 5C).

Considering the crucial role exerted by LIG3 in Alt-NHEJ, we assessed Alt-NHEJ repair activity after RACGAP1 knockdown using the EJ2-GFP assay. Indeed, we observed a significant decline of Alt-NHEJ repair activity in GBC cells depleted of RACGAP1 as compared to controls (Figure 5D).

Moreover, our results showed that augmented LIG3 expression partially reversed the effect of RACGAP1 knockdown on cell viability and colony formation (Figure 5E). Co-transfection of RACGAP1 siRNA with LIG3 expression vector rescued GBC cells from γH2A.X accumulation, activation of the DDR and caspase-dependent apoptotic signaling pathway (Figure 5F), indicating that siRACGAP1-mdediated dysfunctional DNA repair, subsequent induction of DDR and apoptosis are partly due to LIG3 suppression. Interestingly, we also observed that the Poly(ADP-ribosyl) modification (PARylation) increased after RACGAP1 knockdown (Figure 5C). Poly(ADP-Ribose)polymer (PAR) formation will lead to cell apoptosis directly 26, 27, which may be another reason for viability inhibition of GBC cells after RACGAP1 knockdown. LIG3 overexpression antagonized increased PAR signal triggered by RACGAP1 suppression (Figure 5F). These findings indicated that augmented PARP1 catalytic activity triggered by RACGAP1 suppression is the consequence of LIG3 downregulation. We validated the *in vivo* effect of LIG3 upregulation on RACGAP1-depleted GBC cells as well. Tumor growth inhibition caused by RACGAP1 knockdown could be partially reversed by LIG3 overexpression (Figure S6), and we assessed the expression of γH2A.X, PAR and apoptotic signal in the subcutaneous tumors through IHC. As depicted in Figure 5G, overexpression of LIG3 decreased the levels of aforementioned molecular markers in shRACGAP1 tumors, therefore confirming our *in vitro* data.

### RACGAP1 is dispensable for localization of LIG3 but increases its stability

**Figure 6:**
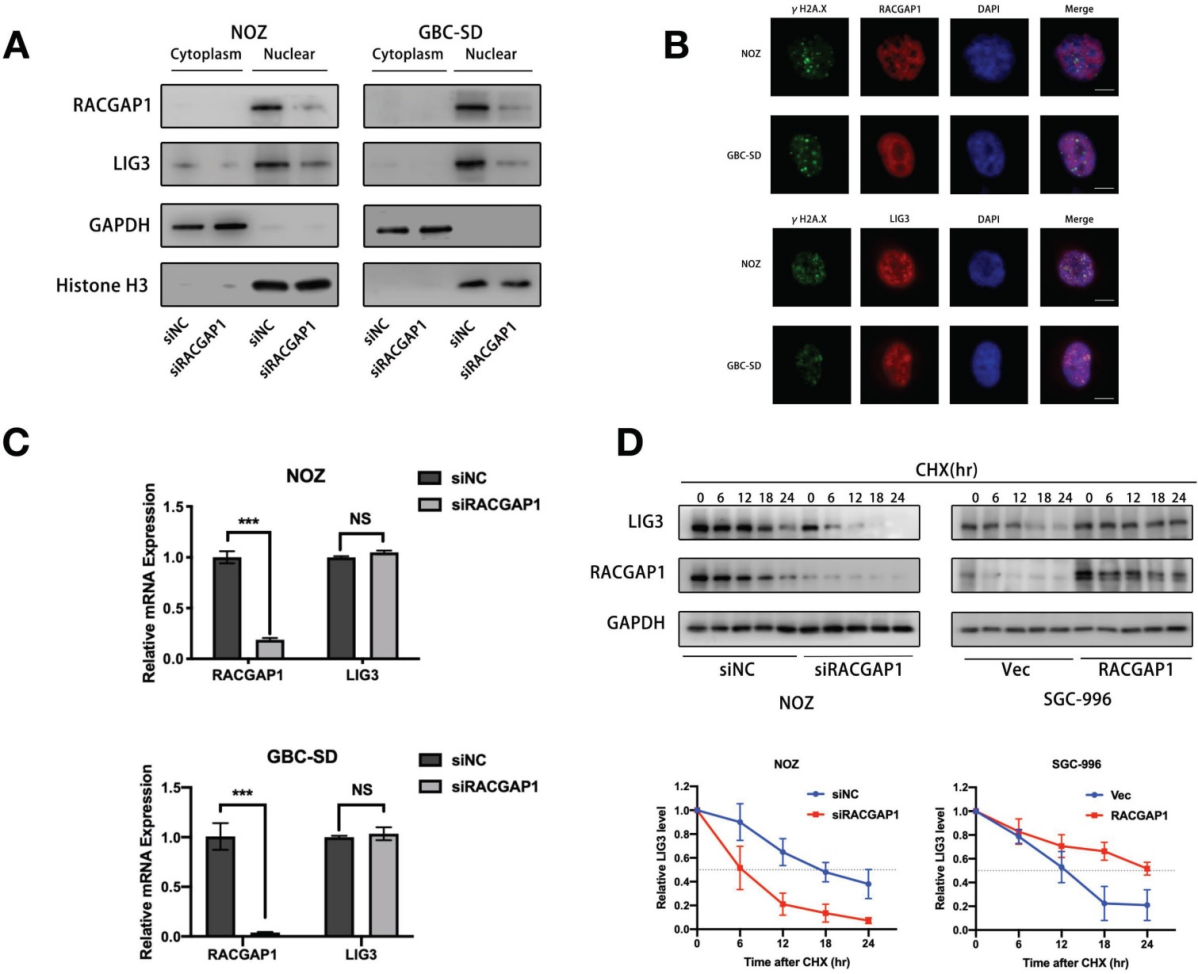
**RACGAP1 is dispensable for localization of LIG3 but increases LIG3 stability.** (A) Subcellular LIG3 expressions in NOZ and GBC-SD cells were assessed using western blotting. (B) IF images of RACGAP1, LIG3, γH2A.X and DAPI in NOZ and GBC-SD cells after etoposide treatment. Scale bars represent 10 μm. (C) LIG3 mRNA expressions were detected by qRT-PCR in NOZ and GBC-SD cell lines transfected with siNC or siRNA against RACGAP1. (D) LIG3 protein level analyzed by western blotting after knocking down or overexpressing RACGAP1 and treatment with CHX at the indicated times. NS, no significance; ***P < 0.001.

It is known that the majority of DNA damage signaling exists in the nucleus. Previous reports underscore the ability of RACGAP1 to function as a nuclear chaperone 28. We therefore examined the effect of RACGAP1 on the nuclear localization of LIG3. However, knockdown of RACGAP1 did not suppress LIG3 translocation from the cytoplasm to nucleus, as shown in our western blotting experiments (Figure 6A). Then we administered etoposide, a DNA-damaging agent, to GBC cells in order to induce DSBs. IF assays showed that the RACGAP1 protein did not accumulate on the γH2A.X foci after etoposide treatment, whereas a portion of LIG3 localized on γH2A.X foci as described in previous studies 13, 17 (Figure 6B), which suggested that RACGAP1 is not responsible for localization of LIG3 at DSBs, a necessary location of LIG3 repair function.

In order to determine that how RACGAP1 governs LIG3 expression levels, we investigated whether it affected the transcriptional activity or stability of LIG3. Interestingly, we failed to observe any significant changes of mRNA expression which were sufficient enough to decrease LIG3 protein level in cells depleted of RACGAP1 (Figure 6C), suggesting that LIG3 suppression is at the post-transcriptional level. We next examined stability of the LIG3 protein after RACGAP1 downregulation and overexpression. GBC cells were exposed to cycloheximide (CHX), a protein synthesis inhibitor for various times. In RACGAP1 knockdown cells, LIG3 protein levels decreased by nearly 50% within 6 h after CHX treatment in contrast to cells in the control group. The reversed pattern was seen in cells with enhanced RACGAP1 expression, where the half-life of LIG3 proteins was dramatically increased in comparison to the controls (Figure 6D). These observations revealed that RACGAP1 mainly regulates the expression of LIG3 by controlling the stability of the LIG3 protein.

## Discussion

Previous studies have uncovered the oncogenic potential of elevated RACGAP1 in a number of malignant tumors. It appears to be involved in a variety of molecular mechanisms associated with cancer progression. For example, RACGAP1 has been identified as a HCC cell growth promoter that works through promoting cytokinesis as well as suppression of Hippo and YAP pathways 6. It promotes cell proliferation and metastasis through regulation of STAT3 phosphorylation in bladder cancer cells 7. In basal-like breast cancer, depletion of RACGAP1 impaired cell growth via partly resulting from p21-induction and onset of senescence 9. A recent study on breast cancer revealed that RACGAP1 promotes mitochondrial dynamic driven metastasis through recruiting ECT2 and subsequently activating ERK-DRP1 pathway 29. Here, we found that RACGAP1 expression was upregulated in human GBC tissues. The elevated level of RACGAP1 correlated to the clinical progression of GBC including tumor size, tumor invasion, and a poorer overall survival. Moreover, we verified that RACGAP1 downregulation induces GBC cells apoptosis and cycle arrest. These findings suggest an important role of RACGAP1 in the pathogenesis and progression of GBC.

Our results confirmed that LIG3 is a downstream factor that interacts with RACGAP1 and is subjected to its regulation at the protein level. LIG3 has been found overexpressed in cancers, and it is regarded as a biomarker for Alt-NHEJ addiction for DNA damage repair 15, 30. DNA damage is able to initiate ATR-mediated CHEK1 and ATM-mediated CHEK2 activation 31. Both CHEK1 and CHEK2 activation can induce cell cycle arrest to allow time for DNA repair to take place. On the other hand, accumulation of irreparable DNA damage also leads to caspase-dependent cell apoptosis through the activation of CHEK1 and CHEK2 32. In our study, we observed a significantly activation of ATM, ATR and their targets after RACGAP1 inhibition, which can be rescued through enhanced LIG3 expression. Therefore, cancer cell cycle arrest and apoptosis that occur in the presence of RACGAP1 knockdown is partly due to failure of DNA damage repair induced by LIG3 suppression.

Little is known regarding the DNA damage repair mechanism in GBC. Most of current studies only indicate that mutations or ectopic expression of certain DDR gene may have a significant effect on chemotherapy response in GBC 33, 34, 35. Major DNA lesions of cellular genomic DNA include the apurinic/apyrimidinic sites (AP), strand cross-linking, bulky lesions, mismatch, single-stranded breaks(SSBs) and DSBs. DSBs are critical DNA lesions which impart severe consequences if left unrepaired, often resulting in cell death 11. Sufficient evidence has proved LIG3 to be a pivotal component in the Alt-NHEJ repair pathway which typically occurs as a proper response to DNA DSBs 13, 36. Compared to other repair pathways (HR and C-NHEJ) for DSBs, Alt-NHEJ is more error-prone, consequently genomic instability and chromosomal aberrations, which contributes to mutagenesis and carcinogenesis 37, 38. Overactive Alt-NHEJ repair system triggered by LIG3 upregulation has been identified to be a significant causative factor for several malignant tumors 15, 16. We observed a reduction in efficiency of Alt-NHEJ after RACGAP1 knockdown in GBC cells. Thus, the Alt-NHEJ repair pathway may impart significant functions in the promoting effect of RACGAP1 on GBC.

Poly(ADP-ribosyl) modification (PARylation) of proteins is an important process executed mainly by PARP1 and it is also an apical part of DNA damage response. However, excessive PARylation of nuclear proteins directly promotes cell apoptosis which is termed parthanatos (PARP1-dependent cell death) 27, 39. It is regarded as a novel form of cell programmed death that is distinct from cell necrosis and caspase-dependent apoptosis 26. PARP1 binding domain (zf-PARP) is present on LIG3. Free PARP1 dissociated from LIG3/PARP1 complex could enhances PARylation of nuclear proteins and further parthanatos 17. We found that PAR signal increased after RACGAP1 knockdown in GBC cells, which was reversible in the context of LIG3 overexpression. The explanation for these results is likely to be that the reduction of LIG3 proteins due to RACGAP1 knockdown impedes LIG3/PARP1 complex formation, which allows more liberated PARP1 to catalyze PARylation in nuclei. Taken together, increased apoptotic events observed in flow cytometry analysis after RACGAP1 downregulation may be attributed to both defective DNA repair and excessive PARylation. We concluded that RACGAP1 may contribute to GBC pathology via LIG3-dependent DNA repair system and deregulated PARylation.

As the feature originally discovered, the activity with Rho GTPases is one of the main concerns of RACGAP1 researches, whereas RACGAP1 does not always rely on its Rho GTPase to regulate cellular responses. Yamada T et al. found that RACGAP1 controls cytokinesis as a scaffold independent of its GAP activity in B lymphocytes 40. A study showed that RACGAP1 plays a key role in controlling the growth and differentiation of hematopoietic cells through mechanisms other than regulating Rac GTPase activity 41. Our observations noted that RACGAP1 regulates LIG3 expression in a manner independent of RhoA GTPase in GBC cells. These findings suggested that RACGAP1 is a multifunctional protein that deserves further exploration.

Our study also demonstrated that RACGAP1 regulates LIG3 stability. Yang Yu et al. indicated that POT1 promotes LIG3 proteasomal degradation through DDR-associated kinase activation 42. LIG3 functions as a PARP1-binding nuclear protein that is also subjected to PARylation by PARP1. RNF146, an E3 ubiquitin ligase, ubiquitinates DNA repair proteins including the LIG3 protein through recognition of its PAR chain 43, 44. The above lines of evidence indicate that LIG3 degradation is a complex process involving various factors. The specific mechanism by which RACGAP1 affects the stability of LIG3 protein in GBC cells requires further study.

Although we found that RACGAP1 interacts with LIG3 and regulates LIG3 mediated DNA repair, we did not observe that RACGAP1 localizes at the DSB sites. The specific binding domains between RACGAP1 and LIG3, the interactions between LIG3 and DNA break ends or other DNA repair molecules may be the factors affecting the localization of RACGAP1 at DNA break sites, because the overlaps among these domains or allostery may lead RACGAP1 to be detached from LIG3. This speculation needs to be verified in further investigation. Here we considered that RACGAP1 regulates Alt-NHEJ repair mainly through maintaining high levels of LIG3 protein to repair DSBs, while it itself is not involved in the process of LIG3 localization and repair at the break ends of DNA strands.

Biliary tract stone formation involves long-term oxidative stress and chronic inflammation, which also belongs to causative factors of DNA damage 45. The genomic integrity of patients with gallstones suffers from more frequent and fierce attacks, and compensatory changes may occur in DNA repair system of these cases. Previous study revealed that DNA repair genetic variants are relevant with the risk of developing biliary tract cancer and stones 46. Our data showed that RACGAP1 expression correlates to the presence of gallstones in GBC patients, and RACGAP1 is involved in the LIG3-dependent DNA repair pathway. These findings may provide clues as to why gallstone formation is a putative high-risk factor for the development of GBC.

To conclude, our study shows, for the first time, the tumor-promoting role of RACGAP1 in GBC which is partially mediated by binding to and stabilizing LIG3. Our findings add novel description to the existing function database of RACGAP1 and provide new insights into the mechanisms underlying GBC pathogenesis and progression, which may be useful for early diagnosis and development of therapeutic targets.

## Supplementary Material

Supplementary figures and tables.

## Figures and Tables

**Table 1:** Immunohistochemical analysis of RACGAP1 in GBC and cholecystitis tissues

Group	No. of cases	RACGAP1 expression				*P* value
		Negative (0)	Weak (1-2)	Moderate (3)	Strong (4-6)	
GBC	50	2	11	5	32	**<0.001**
Cholecystitis	50	7	32	7	4	

Bold values indicate statistical significance, *P* < 0.05.

**Table 2:** Association of RACGAP1 expression with the clinicopathological characteristics of GBC

Characteristic	No. of cases	RACGAP1 expression		Chi-square	*P* value
		High	Low		
**Age**					
<60	17	13	4	0.082	0.775
≥60	33	24	9		
**Sex**					
Male	22	16	6	0.033	0.856
Female	28	21	7		
**Associated gallstone**					
Present	**32**	**27**	**5**	**4.973**	**0.026**
Absent	**18**	**10**	**8**		
**Histology differentiation**					
Well or moderate	24	17	7	0.241	0.624
Poor	26	20	6		
**Tumor size (cm)**					
<3	**24**	**14**	**10**	**5.888**	**0.015**
≥3	**26**	**23**	**3**		
**Tumor invasion (AJCC)**					
Tis-T2	**17**	**9**	**8**	**5.937**	**0.015**
T3-T4	**33**	**28**	**5**		
**Lymph node metastasis**					
Present	29	22	7	0.124	0.724
Absent	21	15	6		
**TNM stage (AJCC)**					
0-II	14	9	5	0.954	0.329
III-IV	36	28	8		
Total	50	37	13		

Bold values indicate statistical significance, *P* < 0.05.

## Abbreviations

RACGAP1: Rac GTPase activating protein 1
GBC: gallbladder cancer
DDR: DNA damage repair
LIG3: DNA ligase 3
PARylation: Poly(ADP-ribosyl) modification
PAR: Poly(ADP-Ribose)polymer
GAPs: GTPase activating proteins
DSBs: DNA double-strand breaks
Alt-NHEJ: alternative-non-homologous end joining
HR: homologous recombination
C-NHEJ: classic-non-homologous end joining
OS: overall survival
HGEpC: human gallbladder epithelial cells
