# Supplementary material for: Rac GTPase activating protein 1 promotes gallbladder cancer via binding DNA ligase 3 to reduce apoptosis
Source: Int J Biol Sci. 2021 May 27;17(9):2167–80. doi: 10.7150/ijbs.58857 (PMC8241731; doi:10.7150/ijbs.58857)
Supplement: Supplementary file 1 — Supplementary figures and tables. [file ijbsv17p2167s1.pdf]

Supplementary materials

Supplementary figures:

Figure S1. qRT-PCR analysis of RACGAP1 mRNA expression in both NOZ and GBC-SD cell lines which were transfected with RACGAP1 siRNA and in SGC-996 cell line with RACGAP1-expression vector. ****P* < 0.001.

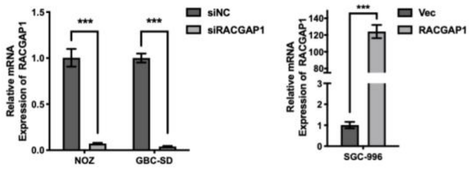

Figure S2. Effect of RACGAP1 overexpression on SGC-996 cell cycle were assessed using flow cytometry. NS, no significance.

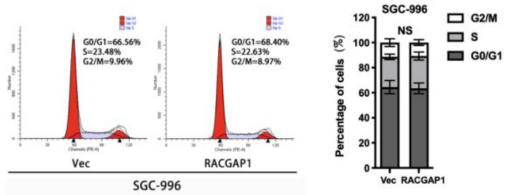

Figure S3. RACGAP1 stably silencing and control NOZ cells were injected subcutaneously into nude mice. Images of tumors from both groups were presented.

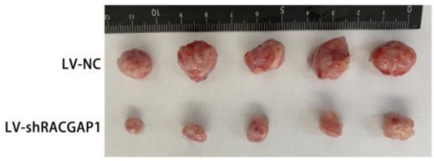

Figure S4. Endogenous Co-IP between RACGAP1 and its potential interaction proteins (TOP2A, KIF2B and PRC1).

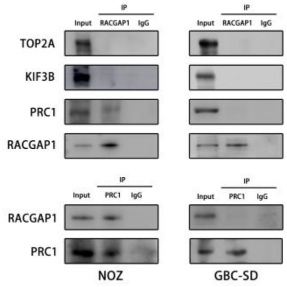

Figure S5. (A) Effects of PRC1 knockdown on NOZ cells viability detected by CCK-8 assays. (B) The levels of apoptosis related proteins (PARP1, c-PARP1, caspase3 and c-caspase3) and γH2A.X in GBC cells after LIG3 or PRC1 knockdown. **P* < 0.05.

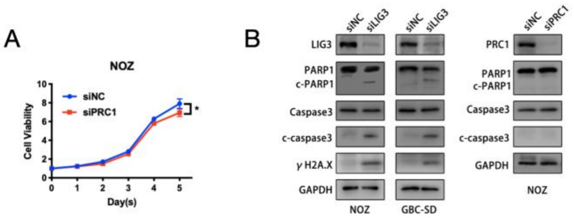

Figure S6. Effect of RACGAP1 on the growth of subcutaneous tumors constructed by injecting NOZ cells transfected with lentiviruses as indicated. Tumor growth are depicted in the form of a line chart. Weight of tumors are depicted using scatterplots. ***P*< 0.01, ****P* < 0.001.

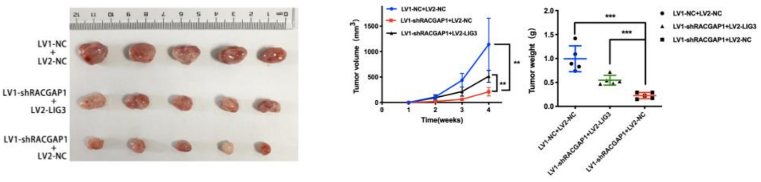

Supplementary tables:

Table S1. siRNA sequences

Gene	siRNA		Sequence
RACGAP1	#1	Sense	5′-GCUGAAGCAUGCACGUAAdTdT-3′
		Antisense	5′-AUUACGUGCAUGCUUCAGdTdT-3′
	#2	Sense	5′-CCCUGGACCUGUAAAGAAAdTdT-3′
		Antisense	5′-UUUCUUUACAGGUCCAGGGdTdT-3′
	#3	Sense	5′-CUCCAUUGUUGUGCAUUGUdTdT-3′
		Antisense	5′-ACAAUGCACAACAAUGGAGdTdT-3′
LIG3	#1	Sense	5′-GGGAAGAGCUGGAAGAUAAdTdT-3′
		Antisense	5′-UUAUCUUCCAGCUCUUCCCdTdT-3′
	#2	Sense	5′-GCAUCAUCAGGUUGAUCAAdTdT-3′
		Antisense	5′-UUGAUCAACCUGAUGAUGCdTdT-3′
	#3	Sense	5′-GCAUGUUCUCUGAGAUCAAdTdT-3′
		Antisense	5′-UUGAUCUCAGAGAACAUGCdTdT-3′
PRC1	#1	Sense	5′-GAAGCUACUUCAAGAGCAAdTdT-3′
		Antisense	5′-UUGCUCUUGAAGUAGCUUCdTdT-3′
	#2	Sense	5′-GCUGUUUACUCAUACAGUAdTdT-3′
		Antisense	5′-UACUGUAUGAGUAAACAGCdTdT-3′
	#3	Sense	5′-GGAGAAUAUUGCAACACUACAdTdT-3′
		Antisense	5′-UAGUGUUGCAAUAUUCUCCAAdTdT-3′

Table S2. Primer sequences used for RT-qPCR

Gene	Primer	Sequence
GAPDH	Forward	GAAATCCCATCACCATCTTCCAGG
	Reverse	CAGTAGAGGCAGGGATGATGTTC
RACGAP1	Forward	CTATGATGCTGAATGTGCGG
	Reverse	AATCCTCAAAGTCCTTCGCC
LIG3	Forward	GGAGGCAGATAGACACAGTATAGG
	Reverse	GGCACCCACAGCAACTAATTC
